# Corrigendum to “Vitamin D and wound healing: Assessing skin barrier function and implications for chloasma treatment”

**DOI:** 10.1111/iwj.14893

**Published:** 2024-04-29

Chen Q, Liu L, Zhang Y. Vitamin D and wound healing: assessing skin barrier function and implications for chloasma treatment. *Int Wound J*. 2024;21 (1):e14541. doi:10.1111/iwj.14541

In the Abstract and Material and Method sections, three instances of ‘Shanghai’ were incorrect. It should have been ‘Chengdu’.

We apologize for this error.

